# Surface Area Estimation: Replacing the Brunauer–Emmett–Teller Model with the Statistical Thermodynamic Fluctuation Theory

**DOI:** 10.1021/acs.langmuir.2c00753

**Published:** 2022-06-17

**Authors:** Seishi Shimizu, Nobuyuki Matubayasi

**Affiliations:** † York Structural Biology Laboratory, Department of Chemistry, University of York, Heslington, York YO10 5DD, U.K.; ‡ Division of Chemical Engineering, Graduate School of Engineering Science, Osaka University, Toyonaka, Osaka 560-8531, Japan

## Abstract

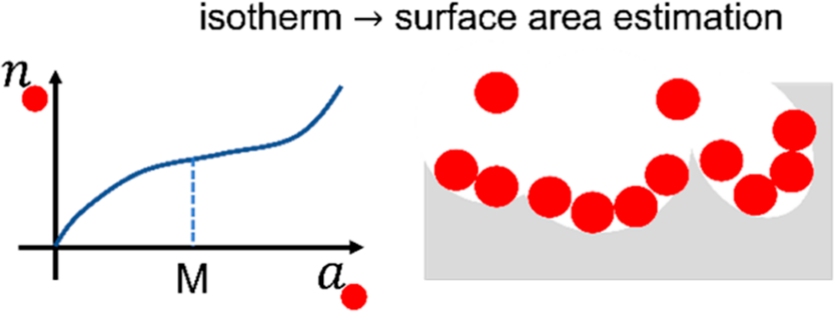

Surface area estimation using the Brunauer–Emmett–Teller (BET) analysis has been beset by difficulties. The BET model has been applied routinely to systems that break its basic assumptions. Even though unphysical results arising from force-fitting can be eliminated by the consistency criteria, such a practice, in turn, complicates the simplicity of the linearized BET plot. We have derived a general isotherm from the statistical thermodynamic fluctuation theory, leading to facile isotherm fitting because our isotherm is free of the BET assumptions. The reinterpretation of the monolayer capacity and the BET constant has led to a statistical thermodynamic generalization of the BET analysis. The key is Point M, which is defined as the activity at which the sorbate–sorbate excess number at the interface is at its minimum (i.e., the point of strongest sorbate–sorbate exclusion). The straightforwardness of identifying Point M and the ease of fitting by the statistical thermodynamic isotherm have been demonstrated using zeolite 13X and a Portland cement paste. The adsorption at Point M is an alternative for the BET monolayer capacity, making the BET model and its consistency criteria unnecessary. The excess number (i) replaces the BET constant as the measure of knee sharpness and monolayer coverage, (ii) links macroscopic (isotherms) to microscopic (simulation), and (iii) serves as a measure of sorbate–sorbate interaction as a signature of sorption cooperativity in porous materials. Thus, interpretive clarity and ease of analysis have been achieved by a statistical thermodynamic generalization of the BET analysis.

## Introduction

Specific surface area is one of the major characteristics of materials as adsorbents.^1−5^ This quantity has been estimated from the adsorption of probe gas sorbates with the help of isotherm models, most commonly by the Brunauer–Emmett–Teller (BET) model.^6,7^ (We use the term “estimation” throughout, appreciating its approximate nature due to the assumptions involved.) Here, the “BET surface area” is defined as the BET monolayer capacity (i.e., “the amount needed to cover the surface with a complete monolayer of atoms or molecules in a close-packed array”^8^) multiplied by the molecular cross-sectional area of the adsorbate.^9,10^ Despite its widespread use,^1−3,11,12^ concerns persist about the validity and accuracy of the BET surface area, which will be summarized below, followed by our approach for clarification and resolution.

### Calculated Surface Area Differs from Sorbate to Sorbate

The BET surface areas are often different from one probe sorbate to another, such as nitrogen and water.^11,12^ According to a systematic comparison for hardened Portland cement pastes, the estimated surface area using nitrogen gas as a sorbate is consistently lower than the one obtained from water vapor.^12^ For food^11^ and microcrystalline cellulose,^13^ the BET surface areas from water can be an order of magnitude larger than their nitrogen-based counterparts. Such a difference has been attributed to a larger molecular size of nitrogen,^12^ to the penetration of water and different states of the sorbed water,^13^ or used as a piece of evidence to question whether the water monolayer really exists.^11^

### Reality of the BET Model Has Been Questioned

**Figure 1:**
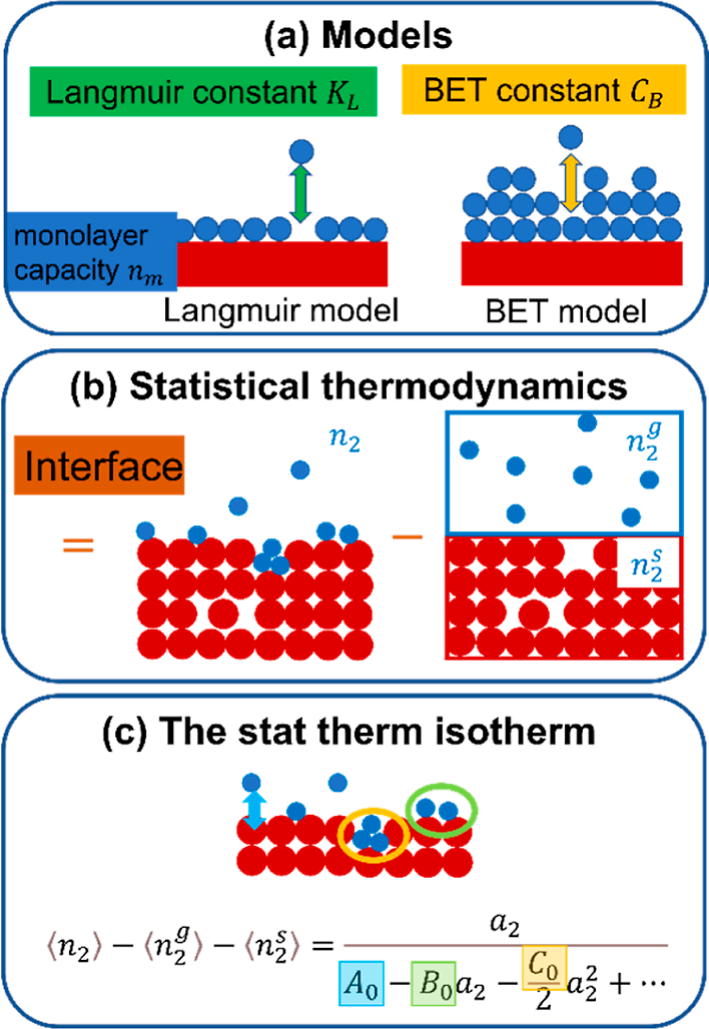
Difference between the previous isotherm models (a) and our statistical thermodynamic approach (b,c). (a) Langmuir model assumes monolayer adsorption on a uniform surface with a binding constant (the Langmuir constant). The BET model assumes each adsorbed molecule as a potential adsorption site for the next layer and neglects interaction between the sorbates in the same layer. The BET constant is related (exponentially) to the difference in binding energies between the first and outer layers. (b) Our statistical thermodynamic approach does not involve any assumptions on binding layers, constants, or the mode of sorbate interaction. Instead, it is based on the difference in sorbate numbers between the system with the interface (left) and the gas and sorbent reference systems (right). (c) Statistical thermodynamic isotherm (eq 7) can be derived by incorporating the sorbate-interface (blue), sorbate–sorbate (green), and sorbate triplet (orange) interactions in the Maclaurin expansion (eq 6). Note that the sorbate–sorbate and sorbate triplet interactions captured using the pair and triplet number correlations are influenced by the presence of the interface (sorbents). Our theory is valid regardless of sorbate and sorbent molecular size and shape. For the precise definitions of *A*_0_, *B*_0_, and *C*_0_, see eqs 8 and 9 and ref (22).

The BET model is based on a set of assumptions that include (1) adsorption on a uniform surface, (2) each adsorbed molecule in a layer is a potential adsorption site for the next layer, (3) no steric limitation on the thickness of the multilayer, (4) no interaction between the molecules in the same layer, and (5) the energy of adsorption on the first layer is higher than the rest (Figure 1a).^3^ However, as has been pointed out, “[t]he BET model appears to be unrealistic in a number of respects. For example, in addition to the Langmuir concept of an ideal localized monolayer adsorption, it is assumed that all the adsorption sites for multilayer adsorption are energetically identical and that all layers after the first have liquid-like properties.”^2^ Furthermore, Rouquerol et al. have even stated that “the BET model does not provide a realistic description of any known physisorption system.”^2^ Hence, the previous discussions on the validity and foundation of the BET surface area have centered around the validity of these assumptions, especially for porous and granular systems.^3,11−13^

### Consistency Criteria are Needed to Remove Unphysical Results from the BET Model

**Figure 2:**
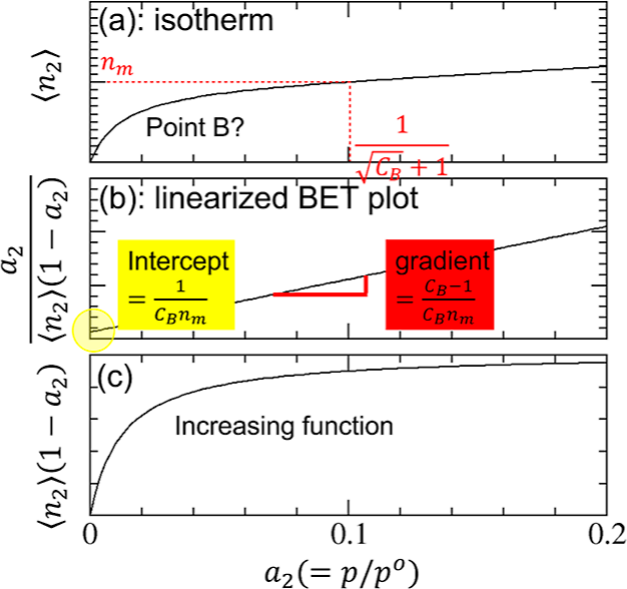
Schematic diagram for a BET isotherm and the consistency criteria. (a) Amount of sorption ⟨*n*_2_⟩ against the sorbate activity *a*_2_ (or equivalently, the relative pressure *p*/*p*^o^) for the BET model with *C*_B_ = 80. Point B, or the knee of the isotherm, is hard to identify by visual inspection. Hence, the consistency criterion D, that is, ⟨*n*_2_⟩ = *n*_m_ at , is employed. (b) Linearized BET plot guarantees that the BET constant is positive (criterion A), and the BET plot is linear at the activity corresponding to the *n*_m_ (criterion C). (c) Increase in ⟨*n*_2_⟩(1 – *a*_2_) at the *a*_2_ corresponding to *n*_m_ (the red dotted line in (a)) satisfies the criterion B.

The BET model has a simple mathematical form; hence, the monolayer capacity and the BET constant can be determined graphically from the linearized BET plot.^3^ However, the BET plot often exhibits linearity over a limited range of sorbate activity (relative pressure).^3,10^ Moreover, identifying the linear region of the BET plot can be subjective.^3,10^ Such long-standing difficulties in fitting the BET model to experimental isotherms have led to the following consistency criteria (Figure 2) that^3,10,14,15^

A. the BET constant *C*_B_ must be positive (Figure 2b);

B. “the application of the BET equation should be restricted to the range where the term *n*(1 – *p*/*p*_0_)continuously increases with [the sorbate activity] *p*/*p*_0_”^10^ (where *n* is the amount of sorption, Figure 2c);

C. the value of the monolayer capacity “should correspond to a relative pressure *p*/*p*_0_ falling within the selected linear region” (Figure 2b);^14^ and

D. “[t]he relative pressure corresponding to the monolayer loading calculated from BET theory [] should be equal to the pressure determined in criterion [C],”^14^ with the tolerance of 20% (Figure 2a).^3,14^

These criteria have been introduced to eliminate unphysical BET parameters, yet their intricacy has made the simple linearized BET plot cumbersome to apply. In addition, the applicability of the criteria has been a matter of debate recently.^14,15^ We will show that such difficulty comes from the restrictive BET model assumption, and its removal makes the analysis of isotherms straightforward.

### Measuring the Monolayer Capacity from the Knee

A core idea of BET is that the monolayer coverage represents the amount of sorption at the knee:^1−3,6,11,12,16^ “If the knee of the isotherm is sharp, the uptake at Point B—the beginning of the middle quasilinear section—is usually considered to represent the completion of the monomolecular layer (monolayer) and the beginning of the formation of the multimolecular layer (multilayer).”^2^ However, because the knee is often ill-defined, it has become usual to derive the capacity from the linearized BET plot. For example, an IUPAC report suggests a method “to obtain by visual inspection the uptake at Point B, which usually agrees with [the monolayer capacity] derived from [the linearized BET plot] within a few percent”,^9^ while admitting that “Point B is not itself amenable to any precise mathematical description, the theoretical significance of the amount adsorbed at Point B is uncertain.”^9^ Even though “the relative pressure [...] for the monolayer capacity can be recalculated from the value of [the BET constant] through the BET equation”^3^ and has been used as a criterion for consistency,^3,15^ its underlying significance beyond its definition has remained unclear. As we will show later, stepping away from the BET formalism allows the direct and unambiguous method for identifying the knee point in a mathematically precise manner with clear physical insights, even for knees that are not sharp, thereby restoring the intuitive idea of the knee to its proper place.

### Applicability of the BET and GAB Models is Much Wider than Their Original Assumptions

Non-planar, granular, and powder systems with moisture *ab*sorption have been modeled routinely by the BET model and by its extension, the Guggenheim–Anderson–de Boer (GAB) model,^17−19^ even though these models were originally derived exclusively for adsorption, assuming planar surfaces with successive adsorption onto multiple layers.^20^ This contradiction was resolved by the current authors using statistical thermodynamics (Figure 1).^21−25^ A general isotherm, which contains the BET and GAB models as its special cases,^22^ has been derived from a Maclaurin expansion of the sorbate–sorbate interaction (quantified via the Kirkwood–Buff integral) at the dilute limit, incorporating up to sorbate pair and triplet contributions (Figure 1b,c). This has fulfilled “the need to examine the limitations of the BET method and in particular to attempt to define the conditions which govern its application”;^2^ the wide applicability of the BET or GAB comes from the sorbate pair and triplet contribution instead of the planar multilayer assumption, rationalizing why the BET and GAB models are widely applicable beyond their original assumptions.^22^ In the current paper, we build on those insights to address the problems with surface area estimation.

### Regional Isotherms

Crucial for BET surface area estimation is the identification of the region of sorbate activity (relative pressure) within which the BET plot is linear. As pointed out by IUPAC, “the range of linearity of the BET plot is always restricted to a limited part of the isotherm – usually not above [*a*_2_] ∼ 0.3”,^26^ and typically the linear region is chosen between 0.05 and 0.3.^3,10^ However, ambiguity persists on how to choose the range of linearity, leading to the multiplicity of the BET parameters.^3^ Moreover, some regions of linearity yield negative monolayer capacity.^3^ This highlights a contradiction: while the BET model is agreed to be applicable to a limited range of sorbate activity (relative pressure), extrapolation to zero activity in the BET plot, beyond this limited range, is indispensable for evaluating the BET parameters.

### Our Strategy

The debate on the foundation and legitimacy of the BET surface area was centered around the validity of the BET model assumptions and the range of sorbate activity (relative pressure) to which they are applicable. Based on our recent clarification on the foundation of the BET model based on statistical thermodynamics,^22^ a new and alternative approach, consisting of the following three steps, is necessary:

I to start from the universal statistical thermodynamic principles of sorption (Figure 1b),
II to translate what the BET monolayer capacity and the BET surface area mean in the language of statistical thermodynamics and molecular interaction (Figure 1c), and
III to overcome the difficulties arising from applying the equation for the entire isotherm to regional isotherm data, namely, to eliminate the need for extrapolating to a zero activity limit.

These steps will lead to a redefinition of interfacial coverage and sorbate packing in the framework of the statistical thermodynamic fluctuation theory. The new method to analyze isotherms will be more straightforward because the restrictive BET model assumption and the consistency criteria are no longer necessary.

## Theory

### Overview

Here, we outline what will be achieved in this section to address the particular issues of the BET model identified in the Introduction. Each bullet point refers to a subsection within the Theory section.

- A rigorous statistical thermodynamic fluctuation approach to sorption will be presented, linking the gradient of an isotherm to sorbate number fluctuation. This is in contrast to the existing isotherms, such as the BET model, constructed on the assumptions of adsorption sites, adsorption layers, and association constants (Figure 1a). We derive the statistical thermodynamic isotherm via the Maclaurin expansion, incorporating sorbate-interface, sorbate–sorbate, and sorbate triplet interactions (Figure 1b,c).
- Re-interpreting the BET model from the statistical thermodynamic fluctuation theory will be made possible because the BET model is a restricted case of the statistical thermodynamic isotherm. This enables us to attribute a statistical thermodynamic reinterpretation of the BET model constants.
- Fitting an isotherm regionally around an activity of relevance is sufficient for linking an isotherm to fluctuations, in contrast to the BET model, whose parameters are defined down at the zero sorbate activity limit (as will be shown in Results and Discussion).
- The interfacial capacity, as the statistical thermodynamic generalization of the BET monolayer capacity, will be introduced, such that the BET analysis, which has been carried out for systems beyond the BET model assumptions, can be generalized to wider classes of sorption phenomena (in Results and Discussion).

### Rigorous Statistical Thermodynamic Fluctuation Approach for Sorption

#### Fluctuation Theory Links an Isotherm to the Underlying Molecular Interactions

A statistical thermodynamic foundation is indispensable for overcoming the difficulties of BET surface area estimation identified in the Introduction (step I), instead of continuing to examine whether the BET model applies to a particular class of materials. As will be shown below, a statistical thermodynamic reinterpretation of the monolayer capacity and BET constant involves a particularly careful discussion on the low sorbate activity limit. Although our previous theory^22^ is valid at this limit (Supporting Information), a generalization is necessary to prove that we can focus safely on the amount of sorption, instead of the surface excess, even at this limit. Throughout this paper, we denote the sorbent as species 1 and the sorbate as species 2. We start from the generalized Gibbs isotherm, which is valid for any geometry, porosity, or granularity of the interface, regardless of molecular size and shape.^21^ Restricting our consideration to vapor–solid interfaces, we have shown previously that the difference between the ensemble-averaged (denoted by ⟨ ⟩) number of sorbates within the two subsystems of volume *v*, one at the interface, ⟨*n*_2_⟩, and another in the vapor (gas) and solid reference phases, ⟨*n*_2_^g^⟩ and ⟨*n*_2_^s^⟩, is expressed as^21^
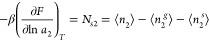
1where *F* is the free energy of the interface (Figure 1b). Equation 1 is applicable regardless of the interfacial geometry and porosity and is valid for adsorption and absorption alike.^21,22^ How the surface excess, *N*_s2_ = ⟨*n*_2_⟩ – ⟨*n*_2_^g^⟩ – ⟨*n*_2_^s^⟩, depends on the sorbate activity *a*_2_ can be characterized through its derivative
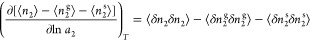
2in terms of the difference in sorbate–sorbate number correlations between the interface, ⟨δ*n*_2_δ*n*_2_⟩, and the vapor and solid reference systems, ⟨δ*n*_2_^g^δ*n*_2_^g^⟩ and ⟨δ*n*_2_^s^δ*n*_2_^s^⟩, with δ*n*_2_ ≡ *n*_2_ – ⟨*n*_2_⟩, δ*n*_2_^g^ ≡ *n*_2_^g^ – ⟨*n*_2_^g^⟩, and δ*n*_2_^s^ ≡ *n*_2_^s^ – ⟨*n*_2_^s^⟩ defined as the deviations from the mean sorbate numbers, respectively. (The background material for the derivation of eq 2 from eq 1 can be found, e.g., in p 129, eq 25.19 of ref (27)). How the isotherm depends on *a*_2_, according to eq 2, is governed by the excess number fluctuation.

#### Statistical Thermodynamic Isotherm Can Be Derived from Sorbate Number Fluctuations

Our next goal is to translate the BET monolayer capacity (the key quantity from which the BET surface area is calculated) into the language of rigorous statistical thermodynamics (step II in Introduction). To do so, we start from the following relationship which can be derived from eq 2 as the generalization of our previous paper,^22^ as
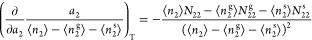
3Here, the sorbate excess number around a probe sorbate, *N*_22_, together with the corresponding quantities for the reference states (*N*_22_^g^ and *N*_22_^s^) have been introduced and defined as^21,22^
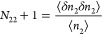
4(In deriving eq 3, the number–number correlations appearing from differentiating the numbers using eq 2 are replaced via eq 4 by the excess numbers.) The excess number is used universally in solutions,^28−30^ interfaces,^21−23^ surfactants,^31^ nanoparticles,^32^ and confined systems.^33^ The utility of eq 3 can best be seen in its following integrated form
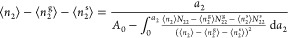
5where *A*_0_ is a constant of integration (whose physical interpretation will be clarified below). Introducing the Maclaurin expansion of eq 3
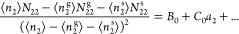
6and combining it with eq 5 yields the following general isotherm (Figure 1c)
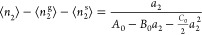
7Equation 7 is our statistical thermodynamic isotherm. Our previous theory^21,22^ results from ⟨*n*_2_⟩ – ⟨*n*_2_^g^⟩ – ⟨*n*_2_^s^⟩ ≃ ⟨*n*_2_⟩ as shown in the Supporting Information. We will later demonstrate that eq 7 contains the BET model as its special case. Here, we show that the parameters have a clear physical meaning. First, we will establish how *A*_0_ is related to sorbate–surface interaction at the *a*_2_ → 0 limit (Figure 1c). This can be achieved by the relationship between *a*_2_ and the gas-phase density, *c*_2_^g^, via *a*_2_ = *c*_2_^g^/*c*_2_^o^, with *c*_2_^o^ being the vapor concentration in the saturated vapor through which *A*_0_^–1^ can be related to the surface–sorbate (or sorbent–sorbate) Kirkwood–Buff integral, *G*_s2_, as^22^
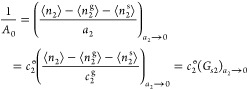
8with the subscript denoting the *a*_2_ → 0 limit. Here, a positive surface–sorbate (or sorbent–sorbate) Kirkwood–Buff integral signifies the accumulation of sorbates at the interface compared to the vapor phase, whereas the negative value signifies their depletion at the interface. (The convergence of *A*_0_ will be shown by its correspondence to the BET parameters in the next paragraph, as well as a careful discussion on the limiting behavior in the Supporting Information.) Second, the parameter *B*_0_ is linked to the excess sorbate–sorbate number fluctuation at the *a*_2_ → 0 limit, as can be seen straightway from eq 6
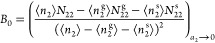
9We emphasize that the sorbate–sorbate number fluctuation here already incorporates the influence by the presence of the interface (sorbent) because the sorbent has already been incorporated in carrying out the ensemble averaging in calculating ⟨*n*_2_⟩ and *N*_22_ (Figure 1c). In our discussion below, the statistical thermodynamic interpretations of the coefficients *A*_0_ and *B*_0_ will play a central role in clarifying the physical meaning of the BET model (Figure 1c). Although *C*_0_ is important for describing some limitations of the BET model, the expression for the coefficient *C*_0_ is complex, involving the sorbate triplet correlation as shown before^22^ and is not discussed further in this paper.

### Interpreting the BET Model from the Statistical Thermodynamic Fluctuation Theory

Based on our generalized theory of sorption which is capable of describing the zero sorbate limit, here we show that the statistical thermodynamic isotherm (eq 7) has the mathematical form (i.e., the quadratic function of *a*_2_ in the denominator and *a*_2_ in the numerator) that contains the Langmuir,^34^ BET,^6^ and GAB^17−19^ models as its special cases.^22^ This makes it possible to translate the “monolayer capacity” *n*_m_ and “the BET constant” *C*_B_ of the BET model (Figure 1a) into statistical thermodynamics (Figure 1b,c, step II in Introduction). The BET model has the following functional form:
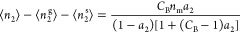
10aComparing eqs 7 and 10a leads to the following correspondence between the BET parameters and statistical thermodynamics
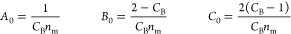
10bOr equivalently
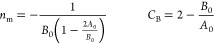
10cThus, the BET model parameters have been given a statistical thermodynamic interpretation by eq 10c, in combination with eqs 8 and 9. Based on this new interpretation, we will later clarify what the BET “monolayer capacity” signifies in the language of statistical thermodynamics (see Results and Discussion).

### Regional Isotherm Fitting Around an Activity of Relevance is Sufficient for Linking an Isotherm to Fluctuations

So far, we have compared the BET model (eq 10a) with the statistical thermodynamic isotherm (eq 7) over the entire range of activity (*a*_2_). However, the protocol for the BET surface area calculation involves the identification of the *a*_2_ range in which the BET model fits the experimental isotherm data.^9^ Such a fitting region is to be found typically between *a*_2_ = 0.05 and 0.30, with the applicability of BET evidenced by the linearity of the BET plot.^9^ Hence, it is necessary to adapt our theory to regional isotherm fitting; that is, fitting over a small region of *a*_2_ around a reference (*a*_2_ = *a*_r_) instead of the global fit over all *a*_2_. To do so, the Maclaurin expansion in eq 6 is modified as
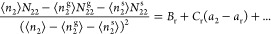
11aand the integration of eq 5 is changed to
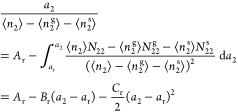
11bwith the constants *A*_r_, *B*_r_, and *C*_r_ defined at *a*_2_ = *a*_r_. *A*_r_ is now linked to the surface–sorbate Kirkwood–Buff integral at *a*_2_ = *a*_r_
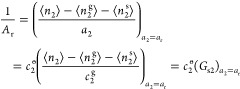
11cand *B*_r_ is related to the sorbate number fluctuations at *a*_2_ = *a*_r_ as
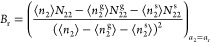
11dAlso, *C*_r_ involves ternary number correlations. Defining the isotherm parameters regionally at *a*_2_ = *a*_r_ will help overcome some of the historical difficulties surrounding the BET analysis of isotherms (Results and Discussion). The application of this approach will be simplified in the next paragraph.

### Interfacial Capacity as the Statistical Thermodynamic Generalization of the BET Monolayer Capacity

The BET monolayer capacity is a quantity defined under the assumptions of the BET model. Our aim here is to define a statistical thermodynamic quantity, the “interfacial capacity”, as a generalization of the BET monolayer capacity and independent of the BET model assumptions. The key to generalization comes from the statistical thermodynamic translation of the BET model parameters (eqs 10b and 10c) and the IUPAC technical report (“[i]t is now generally agreed that the value of [*C*_B_] rather gives a useful indication of the shape of the isotherm in the BET range. Thus, if the value of [*C*_B_] is at least ∼80, the knee of the isotherm is sharp”^10^), supported also by the NIST recommendation which expresses that “[t]o obtain a reliable value of *n*_m_, it is necessary that the knee of the isotherm be fairly sharp (i.e., the BET constant [*C*_B_] is not less than about 100)”.^35^ Therefore, we can consider *C*_B_ to be large. Under this condition, a combination of eqs 9 and 10b leads to the following relationship
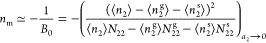
12aEquation 12a can be considered as a special case (*a*_r_ → 0) of the “interfacial capacity” defined as
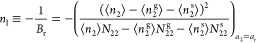
12bwhich is valid both for the regional isotherm around *a*_2_ = *a*_r_ as well as the global isotherm (*a*_r_ → 0). Equation 12b is the statistical thermodynamic generalization of the monolayer capacity. Using eq 3, eq 12b can be rewritten as
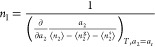
12cEquation 12c allows *n*_I_ to be calculated from any fitting equation. A practical approach is to apply eq 6 instead of eq 11a to carry out regional isotherm fitting within a range of finite *a*_2_. Combining eq 6 with eq 12c, we obtain the following simple expression
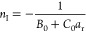
12dIn addition to our isotherm (eq 7), other isotherm models can also be used with eq 12c. The physical meaning of *n*_I_ will be presented in the next section. Thus, we have introduced the “interfacial capacity”, *n*_I_, as a statistical thermodynamic generalization of the BET monolayer capacity *n*_m_. As will be shown in the next section, *n*_I_ will play an important role in understanding interfacial filling.

## Results and Discussion

### Overview

The statistical thermodynamic isotherm will replace the BET model as its model-free generalization.

- Complications due to the BET model assumptions (Figure 1a) will be eliminated, leading to an easier fitting of isotherm data using the statistical thermodynamic isotherm (Figure 1c) without the need for the consistency criteria.
- A new view of sorption will be established based on sorbate–sorbate exclusion, which has been neglected by the BET model.

Based on the demonstrated ease of fitting and interpretation of the statistical thermodynamic isotherm, the BET analysis will be generalized in the framework of the statistical thermodynamic fluctuation theory.

- Problems with the BET monolayer coverage will be identified as being defined inadvertently at zero sorbate activity rather than at full interfacial coverage.
- Interfacial coverage and filling will be redefined statistical thermodynamically as the point of strongest sorbate–sorbate exclusion (Point M).
- Probing interfacial coverage and sorbate packing at Point M will lead to a statistical thermodynamic redefinition of the monolayer–multilayer behavior in adsorption.

This section concludes with a practical summary, a statistical thermodynamic guideline for surface area estimation.

### Fitting Experimental Isotherms Can Be Facilitated by Removing the Restrictive BET Model Assumptions

#### BET Model is a Restricted Case of the Fluctuation Theory

The BET surface area is defined as the product of the BET monolayer capacity (*n*_m_) and the cross-sectional surface (σ_2_).^9,10^ We first focus on the problems associated with the evaluation of *n*_m_ from the experimental isotherm using the BET model. As a first step, we consider an idealized case scenario in which the adsorption isotherm obeys the BET model for the entire *a*_2_ range. As the first step, we show that the BET plot is a restricted case of our statistical thermodynamic isotherm (eq 7), which can be rewritten as
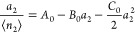
13We emphasize that the three parameters (*A*_0_, *B*_0_, and *C*_0_ with a statistical thermodynamic interpretation in the Theory section) refer to the dilute sorbate limit, *a*_2_ → 0, and are related to the BET parameters via eqs 10b and 10c. In the BET model, the three parameters are not independent; eq 10b reveals the following constraint for the BET model
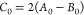
14athrough which eq 13 can be rewritten as
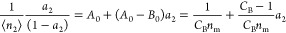
14bwhich is identical to the well-known BET plot shown indeed in Figure 2b.^9,10^ To summarize, the BET plot (eq 14b) contains only two independent parameters compared to three (eq 13) due to the BET model assumption (eq 14a).

#### Force-Fitting the BET Model to Systems beyond the BET Assumptions is the Cause of Difficulties

The BET and Langmuir are highly idealized models. Experimental isotherms often deviate from these models, which poses difficulties to the BET analysis, as discussed in the Introduction. Such a deviation can be captured insightfully by our statistical thermodynamic isotherm (eq 7), which does not involve the constraints imposed by the BET (eq 14a) or Langmuir (*C*_0_ = 0) models. To demonstrate this systematically, we have chosen the following systems as examples.

**Figure 3:**
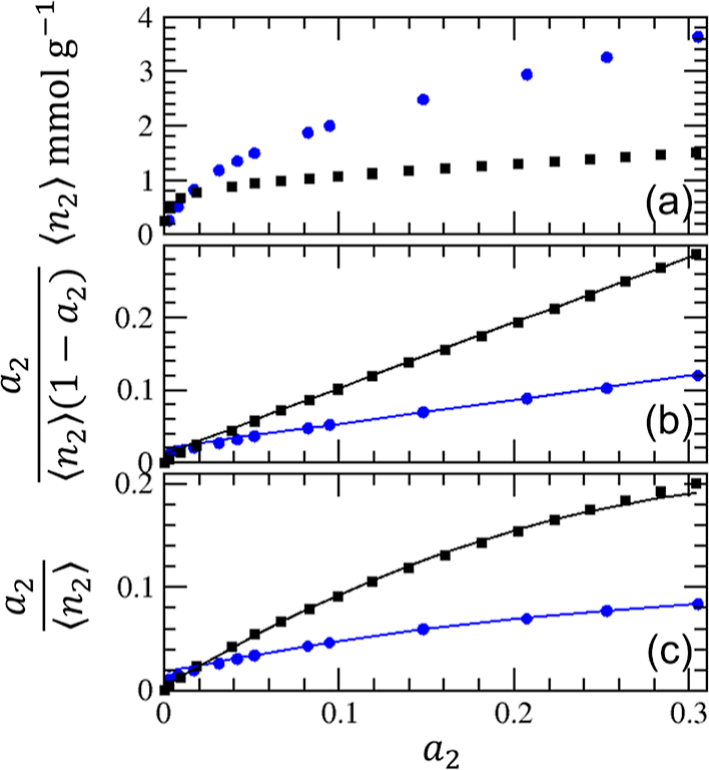
Adsorption of water at 293 K (blue circles) and nitrogen at 77.4 K (black squares) on a Portland cement paste using the data published by Maruyama et al.^36,37^ (a) Adsorption isotherms. (b) BET plot (eq 14b), leading to *C*_B_ = 17.2 and *n*_m_ = 2.86 mmol/g for water and *C*_B_ = 80.6 and *n*_m_ = 1.09 mmol/g for nitrogen, with the resultant BET surface areas from *n*_m_ (196 m^2^/g for water, 106 m^2^/g for nitrogen) consistent with Maruyama et al.^36,37^ (c)  plot (eq 13) with the fitting parameters listed in Table 1.

**Figure 4:**
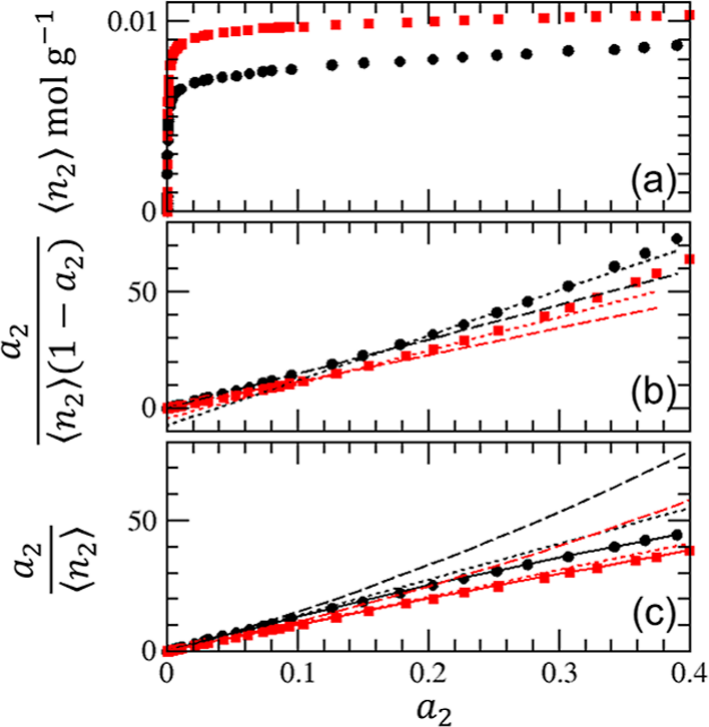
Adsorption of argon on crystalline (red squares) and pelleted (black circles) zeolite 13X using the data published by Pini at 87 K.^38^ (a) Adsorption isotherms. (b) BET plot (eq 14b). Dotted lines: linear fit based on data between *a*_2_ = 0.2 and 0.3 with the unphysical intercepts of  (red) and −7.56 (black), respectively; dashed lines: linear fit based on the data between *a*_2_ = 0.05 and 0.1, with the unphysical intercepts of  (red) and −0.0034 (black). (c)  plot (eq 13) with the fitting parameters listed in Table 1. The dashed and dotted lines were calculated under the BET (*C*_0_ = 2(*A*_0_ – *B*_0_)) and Langmuir (*C*_0_ = 0) constraints.

**Figure 5:**
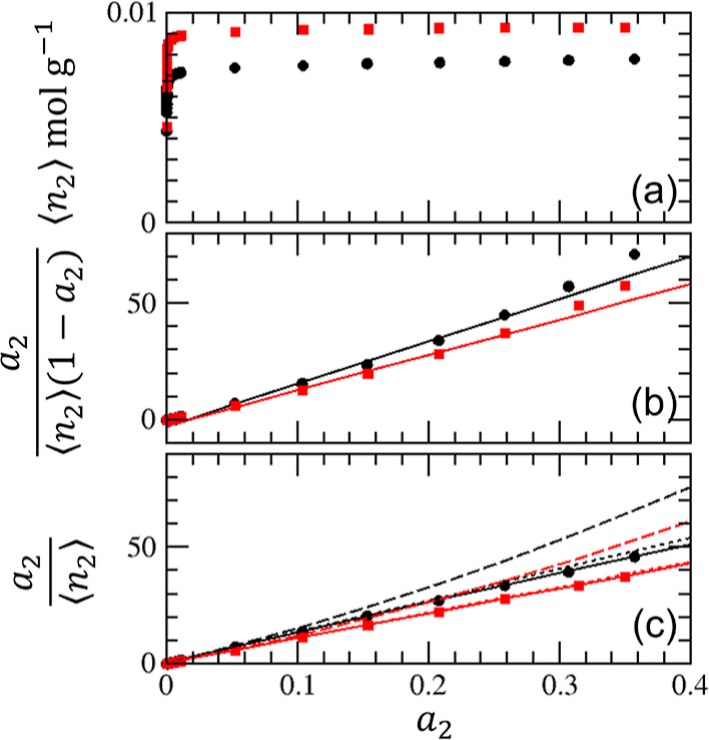
Adsorption of nitrogen on crystalline (red squares) and pelleted (black circles) zeolite 13X using the data published by Pini at 77 K.^38^ (a) Adsorption isotherms. (b) BET plot (eq 14b). Solid lines: linear fit based on data between *a*_2_ = 0.05 and 0.3 with the unphysical intercepts of  (red) and −3.05 (black), respectively. (c)  plot (eq 13) with the fitting parameters listed in Table 1. The dashed and dotted lines were calculated under the BET (*C*_0_ = 2(*A*_0_ – *B*_0_)) and Langmuir (*C*_0_ = 0) constraints.

I The adsorption isotherms of water and nitrogen on a Portland cement paste (Figure 3a) measured by Maruyama et al.^36,37^
II The adsorption of argon and nitrogen on zeolite 13X (Figures 4a and 5a) measured by Pini^38,39^ and chosen by Rouquerol et al.^3^ to illustrate the difficulties of applying the BET analysis to microporous systems.^9,10^

Carrying out the BET analysis via eq 14b (Figures 3b, 4b, and 5b) and determining the parameters for the statistical thermodynamic isotherm via eq 13 (Figures 3c, 4c, and 5c) reveal their varying degrees of closeness to the BET and Langmuir models.

I Both nitrogen and water isotherms for Portland cement can be modeled by the BET model, as evidenced by the linearity of the BET plot (Figure 3b) and by the value of *C*_0_ being not too far from the BET constraint, that is, 2(*A*_0_ – *B*_0_) (eq 14a) as shown in Table 1.
II The BET analysis for zeolite (Figures 4b and 5b) leads to difficulties (as will be discussed below) because the isotherms do not satisfy the condition for the BET model, *C*_0_ = 2(*A*_0_ – *B*_0_). Judging from the value of *C*_0_ (Table 1), the argon adsorption on pelleted samples is neither BET-like nor Langmuir-like. The rest of the isotherms are close to the Langmuir model yet not strictly so because of *C*_0_ ≠ 0 (Table 1).

Given that the BET model is satisfied to a varying degree by the real isotherms, how can we establish a method of surface area estimation that can be used universally instead of force-fitting the BET model to the systems that deviate from it?

**Table 1:** Parameters for the Statistical Thermodynamic Isotherm and a Test of Closeness to the BET (*C*_0_ = 2(*A*_0_ – *B*_0_)) and Langmuir (*C*_0_ = 0) Models^a^

sorbate–sorbent	fitting *a*_2_ range	*A*_0_ g/mmol	–*B*_0_ g/mmol	*C*_0_ g/mmol	2(*A*_0_ – *B*_0_) g/mmol
	eq 13	eq 13	eq 13	eq 13	eq 14a
water/Portland cement^b^	0.04–0.3	1.78 × 10^–2^	3.43 × 10^–1^	8.40 × 10^–1^	7.22 × 10^–1^
N2/Portland cement^b^	0–0.25	4.00 × 10^–3^	1.02 × 10^0^	2.64 × 10^0^	2.04 × 10^0^
Ar/crystalline zeolite 13X^c^	0–0.4	1.04 × 10^–4^	1.03 × 10^–1^	3.46 × 10^–2^	2.06 × 10^–1^
Ar/crystalline zeolite 13X^c^	0.01–0.15	1.33 × 10^–4^	1.04 × 10^–1^	4.38 × 10^–2^	2.08 × 10^–1^
Ar/pelleted zeolite 13X^c^	0–0.4	1.51 × 10^–4^	1.36 × 10^–1^	1.16 × 10^–1^	2.73 × 10^–1^
Ar/pelleted zeolite 13X^c^	0.01–0.15	2.06 × 10^–4^	1.40 × 10^–1^	1.80 × 10^–1^	2.81 × 10^–1^
N_2_/crystalline zeolite 13X^c^	0–0.4	1.17 × 10^–5^	1.09 × 10^–1^	1.01 × 10^–2^	2.18 × 10^–1^
N_2_/pelleted zeolite 13X^c^	0–0.4	1.64 × 10^–5^	1.35 × 10^–1^	4.01 × 10^–2^	2.70 × 10^–1^

a All *R*^2^ values were above 0.9987.

b Data reported by Maruyama et al.^36,37^

c Data reported by Pini.^38^

#### Fundamental Assumptions of the BET Model May be Broken

The first step of surface area determination by BET is to identify the linear region of the BET plot (eq 14b). (Such a process is unnecessary for an isotherm which strictly obeys the BET model, Figure 2b.) The IUPAC guideline advises the linear region to be chosen usually between *a*_2_ = 0.05 and 0.30.^9^ The Portland cement isotherms exhibited good linearity in this *a*_2_ range (Figure 3b). For zeolites, the *a*_2_ regions within this guideline, 0.05 ≤ *a*_2_ ≤ 0.1 and 0.20 ≤ *a*_2_ ≤ 0.3 for Figure 4b and 0.05 ≤ *a*_2_ ≤ 0.3 for Figure 5b, gave negative values for the intercept ( in eq 14b) contradictory to the positive *C*_B_ and *n*_m_ assumed by the BET model. This is because, outside the range of very small *a*_2_ (<0.05), these isotherms violate the consistency criteria listed in Introduction (Table 2). However, how can we analyze isotherms in a simpler manner without the laborious check against the four consistency criteria?

**Table 2:** Determination of Argon and Nitrogen BET Surface Areas of Zeolite 13X Cross-Validated with the Consistency Criteria

sorbate–sorbent	fitting range^b^	data pts	*C*_B_	*n*_m_^c^	BET surface area^d^	(1 – *a*_2_) ⟨*n*_2_⟩ increases until *a*_2_=	*a*_2_ for *n*_m_	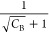
Ar/crystal	0.0003–0.04	21	2.51×10^3^	9.10	775	4.27 × 10^–2^	1.92 × 10^–2^	1.96 × 10^–2^
Ar/pellet	0–0.05	22	1.49×10^3^	6.85	585	7.32 × 10^–2^	2.46 × 10^–2^	2.52 × 10^–2^
N2/crystal	0–0.01	20	7.09×10^4^	8.82	859	1.09 × 10^–2^	3.73 × 10^–2^	3.74 × 10^–3^
N2/pellet	0–0.04	19	4.71×10^4^	7.07	689	9.74 × 10^–3^	4.56 × 10^–3^	4.59 × 10^–3^
criterion^a^			A			B	C	D
the values must be			positive			above the fitting range	within the fitting range	close to the left column

a See the list in the Introduction.

b *R*^2^ values were above 0.9996.

c Units in mmol/g.

d Units in m^2^/g.

#### Removing the BET Restrictions via Statistical Thermodynamics Facilitates Fitting

The difficulty in the BET analysis for zeolite isotherms comes from the restrictive assumption of the BET model (eq 14a) that is not satisfied (Table 1). Therefore, eq 13, free of the BET assumptions, can fit the experimental isotherm over a range of *a*_2_ between *a*_2_ = 0 and 0.4 (Figures 4c and 5c), much wider than the linear regions of the BET plot (Table 2). A straightforward analysis is afforded by the general statistical thermodynamic formula without any constraints on its parameters (eq 13).

### Sorbate–Sorbate Exclusion is the Key to the Statistical Thermodynamic Understanding of Isotherms

#### Our Strategy

Due to the limitations of the BET model, a new theoretical foundation is necessary for surface area estimation. To achieve this goal, our strategy is to fulfill what the BET analysis has aimed to achieve without the restrictions of the BET model. To this end, we will reformulate the key concepts of the BET model (such as the monolayer capacity, the BET constant, and monolayer filling) in the framework of the statistical thermodynamic fluctuation theory based on the correspondence that we have already established (eqs 10b and 10c).

#### Presence of the Interface Affects Sorbate–Sorbate Distribution

We have seen the importance of the sorbate–sorbate excess number *N*_22_ in the Theory section. *N*_22_ is related to the (log–log) gradient of the isotherm as^21,22^
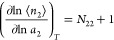
15awhich is a simplified version of eq 2 applicable to common interfaces. Understanding the effect of the interface on sorbate–sorbate interaction can be facilitated by introducing the sorbate–sorbate Kirkwood–Buff integral, *G*_22_, as^21,28,30,40^
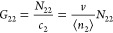
15bwhere *v* is the volume of the interfacial layer (e.g., for a planar, monolayer surface, *v* is simply the product of the monolayer thickness and the interfacial surface area), and *c*_2_ = ⟨*n*_2_⟩/*v* is the concentration of sorbates at the interface. The sign of *G*_22_ is an important signature of sorbate–sorbate interaction; a positive *G*_22_ represents a net sorbate–sorbate attraction, whereas a negative *G*_22_ signifies a net exclusion of sorbates from a probe sorbate.^21,28,30,40^ As will be shown below, *G*_22_ is negative for adsorption obeying the BET model. This is in contrast to the positive sign of *G*_22_^g^, that is, the sorbate–sorbate Kirkwood–Buff integral of the vapor phase evaluated from the experimental virial coefficients^41−43^ (Supporting Information), showing that the presence of the interface influences the sorbate–sorbate distribution, making it different from the vapor phase. In this manner, how the interface (or sorbent) affects the sorbate–sorbate interaction can be captured quantitatively by the Kirkwood–Buff integral.

#### Sorbate–Sorbate Exclusion Determines the BET Constant and Interfacial Capacity

Here, we show statistical thermodynamically that the BET monolayer capacity and the BET constant can only be positive under sorbate–sorbate exclusion, which seems surprising from the common understanding of the BET theory. First, the BET monolayer capacity *n*_m_ (eq 12a) is the *a*_r_ → 0 limit of the interfacial capacity (eq 12b), which can be simplified as
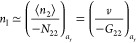
16For the monolayer capacity to be positive, as postulated by the BET model,^3,9,10^*G*_22_ at *a*_r_ → 0 must be negative. This is underscored by the statistical thermodynamic expression of the BET constant simplified in combination of eqs 8, 9, 10c, and 11b as
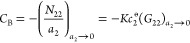
17where *c*_2_^o^ is the concentration of the saturated sorbate vapor, and *K* is the vapor-interface partition coefficient. For the BET constant to be positive (as has been assumed by the BET model), *G*_22_ must again be negative, which signifies sorbate–sorbate exclusion. We emphasize that a positive sign of −*G*_22_, which makes *n*_I_ and *C*_B_ positive, can be interpreted as the measure of volume that a probe molecule occupies at the interface. (Such an interpretation may be most intuitive for thick interfaces, verging onto a bulk liquid, where the positive −*G*_22_ signifies the volume occupied by a sorbate molecule according to the Kirkwood–Buff theory of liquids.^40,44,45^) Therefore, the positive −*G*_22_ as the measure of probe volume at the interface is the generalization of the bulk liquid argument. Such a statistical thermodynamic interpretation is in contrast with the conventional understanding that the BET constant “is exponentially related to the energy of monolayer adsorption.”^10^

### Problems with the BET Monolayer Coverage

#### BET Parameters are Defined at the Dilute Sorbate Limit Far Away from the Monolayer Coverage

The goal of the BET analysis for surface area estimation is to probe “a complete monolayer of atoms or molecules in close-packed array”.^8^ However, both *n*_m_ and *C*_B_ correspond to the dilute sorbate limit (*a*_2_ → 0), as has been shown above (eqs 10b and 10c). This seemingly surprising conclusion can be supported also from a perspective based purely on the BET plot (eq 14b, Figures 4b and 5b). The monolayer capacity *n*_m_ and the BET constant *C*_B_ are evaluated from its gradient () and intercept (), respectively. The intercept, by definition, is the value at *a*_2_ = 0. Therefore, against its claim of capturing monolayer coverage, the monolayer capacity in the BET model is inadvertently defined at the *a*_2_ → 0 limit far away from monolayer coverage.

#### Dilute Sorbate Limit May Be Hypothetical

**Figure 6:**
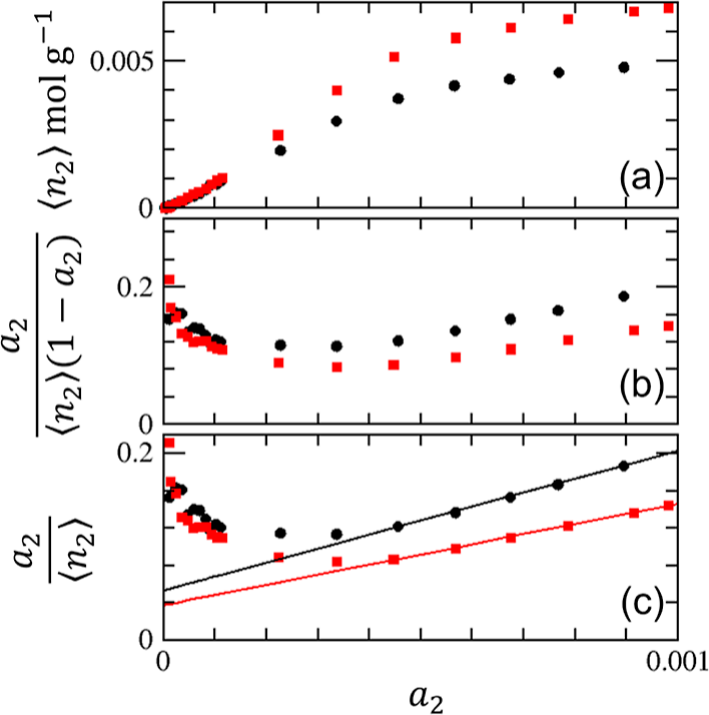
The low *a*_2_ behavior of the argon adsorption on crystalline (red) and pelleted (black) zeolite 13X using the data published by Pini at 87 K.^38^ (a) Adsorption isotherms. (b) The BET plot (eq 14b) which exhibits a negative gradient at *a*_2_ → 0. (c)  plot with the fitting equation (eq 13 with *C*_0_ = 0) using data between *a*_2_ = 4 × 10^–4^ and 1 × 10^–3^.

Here, we demonstrate that the dilute sorbate limit does not correspond to the real sorption behavior at the same limit. (How adsorption at very low *a*_2_ can be measured experimentally^46−52^ is summarized in the Supporting Information). Extrapolation requires a fitting function. However, even with the use of the general polynomial free of BET (eq 13), the extrapolation at *a*_2_ → 0 may still be different from the real system behavior at this limit. For example, at very low *a*_2_, a negative experimental gradient (positive *B*_0_) of the  plot for argon (Figure 6) corresponds (via eq 9) to a positive sorbate–sorbate excess number opposite in sign from the extrapolated behavior. Thus, using the unreal *a*_2_ → 0 extrapolation is problematic for surface area estimation.

### Overcoming the Problems with the BET Monolayer Capacity by Redefining Interfacial Coverage and Filling via Statistical Thermodynamics

#### Point M as the Completion of Interfacial Coverage

**Figure 7:**
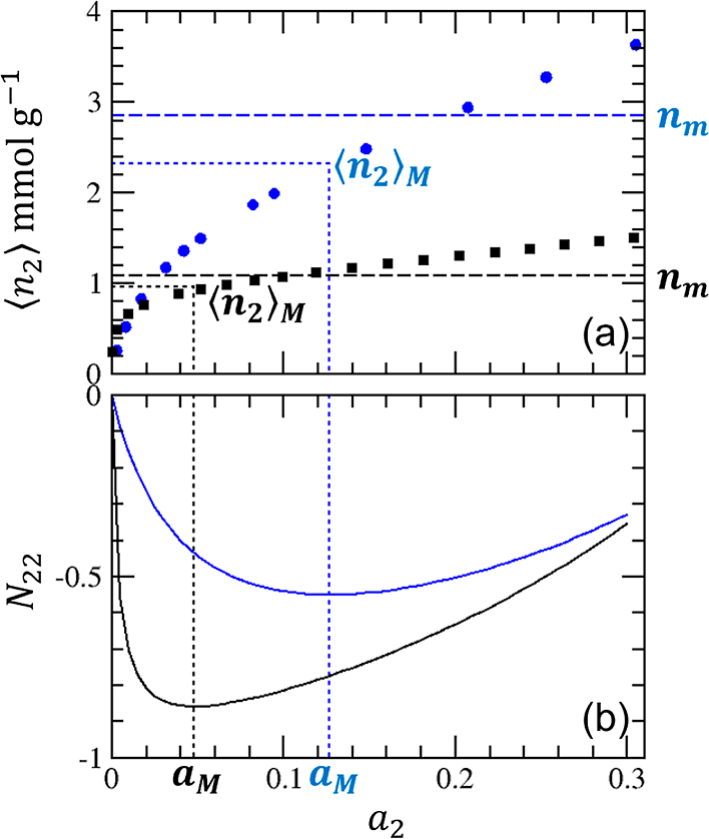
(a) Adsorption of water (blue circles) and nitrogen (black squares) on a Portland cement paste (Figure 3), with the indication of the respective amounts of adsorption at Point M, ⟨*n*_2_⟩_M_ at *a*_2_ = *a*_M_, calculated using eqs 18c and 18d. *n*_m_ is the corresponding BET monolayer capacity determined in Table 2. (b) Excess numbers of sorbates around a probe sorbate, *N*_22_, calculated using eq 18a (with the parameters from Table 1) for water (blue circles) and nitrogen (black squares). Point M, where *N*_22_ is minimum, is calculated using eq 18c.

**Figure 8:**
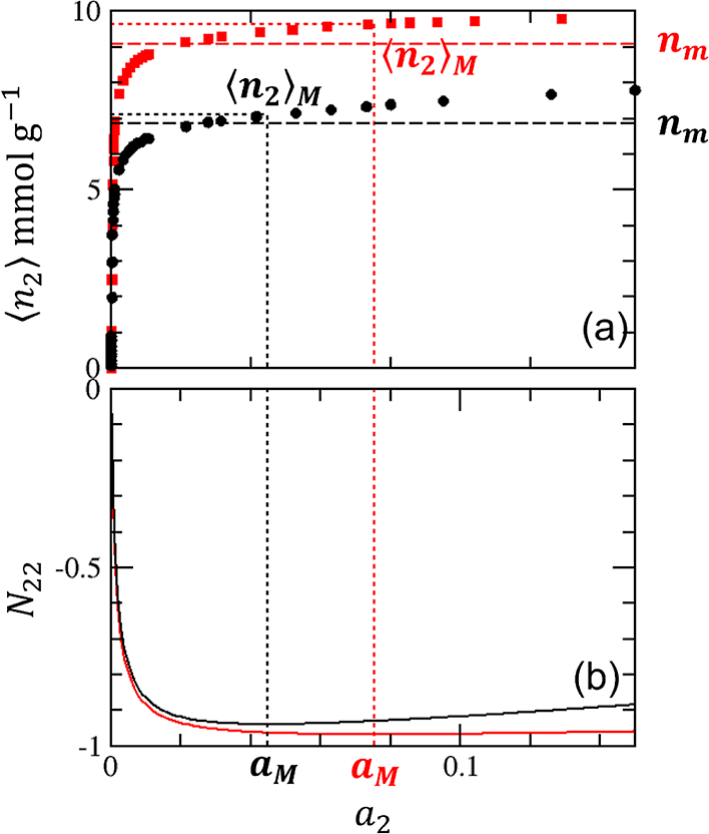
(a) Adsorption of argon on crystalline (red squares) and pelleted (black circles) on zeolite 13X (Figure 4), with the indication of the respective amounts of adsorption at Point M, ⟨*n*_2_⟩_M_ at *a*_2_ = *a*_M_, calculated using eqs 18c and 18d. *n*_m_ is the corresponding BET monolayer capacity determined in Table 2. (b) Excess numbers of sorbates around a probe sorbate, *N*_22_, calculated using eq 18a (with the parameters from Table 1) for crystalline (red squares) and pelleted (black circles) zeolite 13X. Point M, where *N*_22_ is minimum, is calculated using eq 18c.


Our goal is to establish a reliable and facile alternative to BET analysis. The BET analysis has aimed, via *n*_m_, to quantify the amount of adsorption at the knee of the isotherm at which the completion of monolayer filling is assumed to take place.^2,7,9,10^ However, as discussed in the Introduction, since the precise location of the knee (or Point B) is unclear and becomes even more so as *C*_B_ becomes smaller, the BET monolater capacity and the amount of adsorption at Point B may not be reliable quantitative measures. In addition, even though the consistency criteria helped eliminate unphysical results, they have complicated the BET analysis procedure; the root cause of the complication is trying to fit the BET model to the systems that break the BET model assumption (*C*_0_ ≠ 2(*A*_0_ – *B*_0_) (Table 1). To overcome these shortcomings, here, we introduce Point M, at which *N*_22_ takes a minimum, and calculate the amount of sorption at this point. (For an intuitive grasp of Point M, the reader may refer to our results in advance for the Portland cement (Figure 7) and zeolite 13X (Figure 8).) Combining eqs 7 and 15a under ⟨*n*_2_⟩ – ⟨*n*_2_^g^⟩ – ⟨*n*_2_^s^⟩ ≃ ⟨*n*_2_⟩ (Supporting Information), we obtain
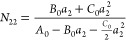
18aAt Point M, (*a*_2_ = *a*_M_),  must be satisfied, which leads to

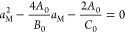
18bSolving eq 18b under *B*_0_ < 0 for sorbate–sorbate exclusion,^22^ we obtain
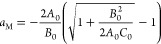
18cConsequently, the amount of adsorption at Point M can be calculated using eqs 7 and 18b as
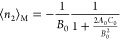
18dRestricting our result (eq 18d) to the BET model using eq 10b, we obtain the following expression for the amount of adsorption at Point M
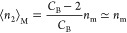
18eThe approximation at the final step is accurate for large *C*_B_. The location of *a*_M_ can also be specified as
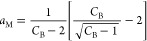
18fEquations 18e and 18f are significant; for large *C*_B_ (as recommended by IUPAC), the monolayer capacity is equivalent to the amount of sorption at Point M for the BET model. Previously,  was identified as the point at which the amount of sorption reaches the monolayer capacity, ⟨*n*_2_⟩ = *n*_m_.^53^ This point has made it to one of the consistency criteria (D) for the BET analysis (see Introduction).^3,15^ Note that *a*_M_ and  are close in values, with less than 12% difference for *C*_B_ > 80, providing a statistical thermodynamic support for the consistency criterion D. However, beyond being the point at which ⟨*n*_2_⟩ = *n*_m_, the physical meaning of the latter has remained unknown, and its applicability has been limited within the BET analysis. In contrast, Point M can be defined for any isotherm.

#### Surface Area Estimation from the Adsorption at Point M

The location of Point M has been defined precisely for the BET model (eq 18f) and even for the statistical thermodynamic isotherm (eq 18c). Therefore, the amount of adsorption at Point M ⟨*n*_2_⟩_M_ is a viable alternative for the estimation of the monolayer capacity, *n*_m_. Indeed, ⟨*n*_2_⟩_M_, calculated via eq 18d, agrees reasonably with the monolayer capacity calculated from the BET analysis (Table 3, which reports a comparison between the statistical thermodynamic surface area ⟨*n*_2_⟩_M_σ_m_ with the BET surface area *n*_m_σ_m_, using the standard values of sorbate cross-sectional areas). Point M values can be calculated precisely by eq 18c, and their location around the knee can be inspected visually by Figures 7 and 8. The clarity in locating Point M contrasts with the inherent ambiguity of Point B.^7^ The clear physical picture underlying the definition of Point M (i.e., minimum *N*_22_) contrasts with the lack of interpretation of  in the criterion D and its strict dependence on the BET model. Moreover, the interfacial capacity *n*_I_ agrees with the amount of sorption at the knee only at *a*_2_ → 0 under the condition that adsorption obeys the BET model down to *a*_2_ → 0. Thus, we propose the amount of adsorption at Point M as the statistical thermodynamic alternative for the BET monolayer capacity.

**Table 3:** Surface Area Estimation via Statistical Thermodynamic Fluctuation Theory Using the Parameters (*A*_0_, *B*_0_, and *C*_0_) in Table 1

sorbate–sorbent	fitting *a*_2_ range	*a*_M_	⟨*n*_2_⟩_M_ mmol/g	(*N*_22_)_M_	stat therm surface area (STSA) m^2^/g	BET surface area m^2^/g (Table 2)
		eq 18c	eq 18d	eq 18a	⟨*n*_2_⟩_M_σ_m_^d^	*n*_m_σ_m_^d^
water/Portland cement^a^	0.04–0.3	0.127	2.32	–0.55	160^d^	196^e^
N2/Portland cement^a^	0–0.25	0.048	0.96	–0.86	94^d^	106^e^
Ar/crystalline zeolite 13X^b^	0–0.4	0.076	9.69	–0.97	829	789
Ar/crystalline zeolite 13X^b^	0.01–0.15	0.075	9.63	–0.97	823	789
Ar/pelleted zeolite 13X^b^	0–0.4	0.049	7.32	–0.96	626	585
Ar/pelleted zeolite 13X^b^	0.01–0.15	0.045	7.10	–0.94	607	585
N2/crystalline zeolite 13X^b^^,^^c^	0–0.4	0.048	9.17	–0.99	895	860
N2/pelleted zeolite 13X^b^^,^^c^	0–0.4	0.028	7.40	–0.99	722	690

a Data reported by Maruyama et al.^36,37^

b Data reported by Pini.^38^

c A narrower fitting range was not feasible due to the sparseness of data around *a*_M_

d We have used the cross-sectional area, σ_*m*_, for argon (0.142 nm^2^) and N_2_ (0.162 nm^2^) taken from the IUPAC recommendations and the one for water (0.114 nm^2^) taken from Odler.^12^

e Compared to 196 and 113 m^2^/g by Aili and Maruyama.^37^

#### Advantage of Statistical Thermodynamic Surface Area Estimation over the BET Model

Point M can be identified simply by fitting the statistical thermodynamic isotherm (eq 10a) around the knee (Figures 7 and 8), without any need for the restrictive BET assumptions, the cumbersome consistency criteria, and the problematic extrapolation to *a*_2_ → 0. Note that a very shallow minimum at Point M for the crystalline zeolite (Figure 8) does not pose any problem because *N*_22_ ≃ −1 means that the isotherm has a near-zero gradient (*N*_22_ + 1 ≃ 0); hence, a small error in positioning Point M does not lead to inaccuracies in the amount of adsorption at that point. Point M, defined as the minimum sorbate–sorbate excess number *N*_22_, has a clear microscopic interpretation. ⟨*n*_2_⟩_M_ is also defined clearly as the net excess sorbate–surface distribution function^21,22^ at this point. Unlike the BET model constants, these distribution functions can be calculated directly from molecular simulation, thereby allowing a direct comparison between simulated and experimental values. Thus, a statistical thermodynamic identification of Point M removes the need for the BET model altogether in surface area estimation.

### Probing Interfacial Coverage and Sorbate Packing Statistical Thermodynamically at Point M

#### Sorbate–Sorbate Interaction as the Measure for Knee Sharpness

**Figure 9:**
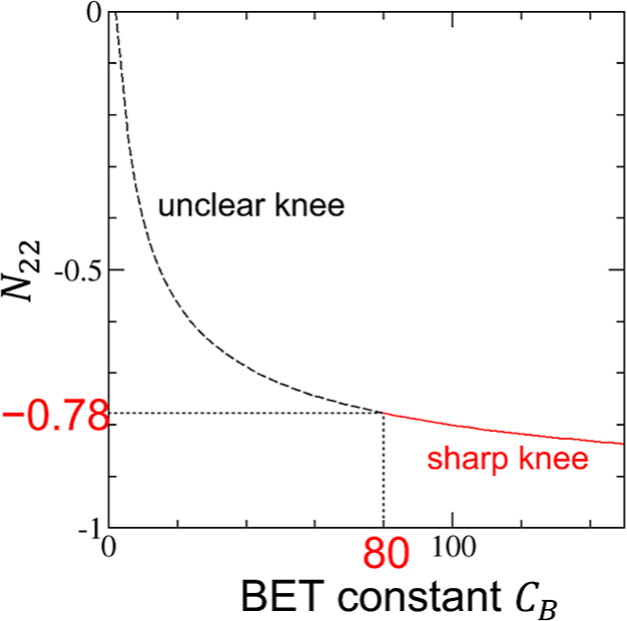
Relationship between the BET constant, *C*_B_, and the excess number of sorbates around a probe sorbate, (*N*_22_)_M_, at Point M plotted using eq 19 derived for the BET model. The IUPAC recommendation that *C*_B_ should be larger than 80 for the clarity of the knee corresponds to *N*_22_ below −0.78. The recommendation based on *N*_22_ can be applied beyond the boundary of the BET model.

In the BET analysis, the BET constant *C*_B_, which determines the shape of an isotherm, is used as a measure for the sharpness of the knee and therefore as evidence for monolayer completion as a prerequisite for surface area determination.^9,10^ IUPAC recommends the BET constant be larger than 80.^9,10^ This recommendation, however, cannot be used for isotherms that do not obey the BET model. Therefore, a new quantitative guideline, independent of sorption models, is necessary. To this end, a relationship between *N*_22_ at Point M and *C*_B_ will be helpful, which can be derived by combining eqs 10b, 18a, 18e, and 18f as
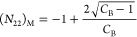
19Using eq 19, a one-to-one correspondence between *C*_B_ and (*N*_22_)_M_ can be established for the BET model (Figure 9). The IUPAC recommendation that *C*_B_ must be larger than 80 is translated as *N*_22_ should be below (i.e., more negative than) −0.78. This criterion also means ⟨*n*_2_⟩_M_/(*n*_I_)_M_ > 0.78, meaning that the amount of sorption at Point M is more than 78% of the interfacial capacity. This new criterion, formulated via *N*_22_, can be applied to any isotherm. The meaning of this criterion will be clarified in the next two paragraphs.

#### Probing the Close Packing of an Interface via the Sorbate–Sorbate Excess Number

To understand the physical meaning of Point M, let us first consider a case in which the gradient of an isotherm is very small at Point M, namely, *N*_22_ ≃ −1. Such a condition is satisfied by the nitrogen and argon adsorption on zeolite 13X (Figure 8) but is different from the Portland cement isotherms (Figure 7, Table 3). *N*_22_ ≃ −1 at Point M is equivalent to

a a very small sorbate number variance, ⟨δ*n*_2_δ*n*_2_⟩ ≃ 0, at Point M, according to eq 4;
b approximately one sorbate molecule in total being excluded around a probe sorbate, *N*_22_ ≃ −1 at Point M, according to eq 4; and
c the amount of adsorption is close to the interfacial capacity at Point M, (*n*_I_)_M_ ≃ ⟨*n*_2_⟩_M_, according to eq 16.

A physical picture of Point M adsorption emerges from (a)–(c) for this case (Figure 8). The interface contains a well-defined number of sorbates because the number fluctuation is small (a). The sorbate molecules are uniformly distributed in the interface because introducing a probe sorbate excludes one sorbate molecule around it (b). Under such a close and uniform packing, the amount of sorption at Point M is close to the interfacial capacity (c). (At the limit of a very thick interface or a bulk sorptive liquid, (a)–(c) can be considered as the conditions for very small compressibility). Therefore, (a)–(c) are the statistical thermodynamic characterizations of very close sorbate packing, most probably due to filling up micropores (which is likely to be the case for zeolite 13X).^23^

#### Sorbate–Sorbate Excess Number May Be a Measure of Monolayer–Multilayer Overlap

Unlike the idealized case in the previous paragraph, the minimum of *N*_22_ is usually above −1 for BET-like systems (Table 3). The existence of a sharp knee corresponds to *N*_22_ below −0.78 (Figure 9). According to IUPAC, the value of *C*_B_ being less than 80 (or, more generally, *N*_22_ above −0.78) is “an indication of a significant amount of overlap of monolayer coverage and the onset of multilayer adsorption”.^10^ This is the case for water adsorption on Portland cement, while nitrogen adsorption behaves closer to the previous paragraph (Figure 7). Compared to *N*_22_ ≃ −1 at Point M, this case shows that

a the sorbate number variance, ⟨δ*n*_2_δ*n*_2_⟩, is larger (eq 4);
b when a probe sorbate is placed, less than one sorbate is excluded (eq 4); and
c the amount of adsorption is smaller than the interfacial capacity at Point M, (*n*_I_)_M_ > ⟨*n*_2_⟩_M_ (eq 16).

Thus, the interface is characterized by the less definite number of sorbates (a) and inhomogeneity in the distribution of sorbates; that is, the presence of a probe molecule (whose center of mass is at rest) makes its vicinity deviate from the sorbate distribution (b). Due to the inhomogeneity and fluctuation at the interface, the amount of adsorption does not reach the interfacial capacity at Point M (c). (Using, as before, the limit of a very thick interface or a bulk sorptive liquid, (a)–(c) correspond to higher compressibility). Such an interfacial behavior is reminiscent of a “significant overlap of monolayer coverage and the onset of multilayer adsorption”^10^ as viewed from the fluctuation theory.

#### Monolayer Coverage May Take Place between the Two Extremes

While small *C*_B_ is the sign of monolayer–multilayer overlap, “[a] high value of [*C*_B_] (say, >∼150) is generally associated with either adsorption on high-energy surface sites or the filling of narrow micropores.”^10^ The recommended values of *C*_B_ between 80 and 150 correspond to the range of *N*_22_ at Point M between −0.78 and −0.84. The BET-based IUPAC guideline was translated to the more universal language of statistical thermodynamics, applicable beyond the bounds of the BET model. However, more investigations are necessary to specify the range of *N*_22_ for monolayer coverage with sufficient clarity for surface area estimation. We have shown that the monolayer–multilayer adsorption mechanism may be operative between the two extremes.

#### Quality of Surface Area Estimation Depends on the Probe Used

The significant difference in surface area between water and nitrogen sorbate probes has been recognized for a long time,^12^ which is true also for the Portland cement (Table 3). However, the quality of surface area determination depends on the probes used. We first note that the minimum *N*_22_ for water is −0.55, while that for nitrogen is −0.86 (Table 3). A larger *N*_22_ means a steeper isotherm gradient; a stronger water–water interaction helps adsorb more water onto the interface. However, according to the correspondence between *N*_22_ and *C*_B_ (Figure 9), water fails the IUPAC recommendation of *C*_B_ > 80 while nitrogen satisfies it. Microscopically, ⟨*n*_2_⟩_M_ = 0.55(*n*_I_)_M_ (eq 16) means that the amount of water adsorption at Point M is only half of its interfacial capacity. Based on the previous paragraph, water adsorption exhibits a significant overlap between monolayer coverage and multilayer adsorption. Consequently, nitrogen seems to be a more appropriate probe than water for surface area estimation in this particular system.

### Statistical Thermodynamic Guideline for Surface Area Estimation

#### Procedure

Here, we summarize the statistical thermodynamic analysis in terms of the following list of procedures for surface area estimation:

1 Fit an experimental isotherm around its knee using eq 13 (Figures 3c, 4c, and 5c, and Table 1).
2 Calculate the location of Point M (*a*_M_) using eq 18c and the amount of adsorption at Point M (⟨*n*_2_⟩_M_) eq 18d (Table 3).
3 Estimate the surface area by multiplying ⟨*n*_2_⟩_M_ by the probe molecule’s cross-sectional area σ_m_ (Table 3). This can be called the statistical thermodynamic surface area (STSA) as an alternative for the BET surface area.

Note that *a*_2_ and ⟨*n*_2_⟩ correspond directly to *p*/*p*_0_ and *n* of the IUPAC notation, respectively. Hence, *a*_M_ and ⟨*n*_2_⟩_M_ are simply *p*/*p*_0_ and *n* at Point M, respectively. Here are the considerations for a sense check:

- the location of *a*_M_ and ⟨*n*_2_⟩_M_ are roughly around the knee of an isotherm (Figure 7a and 8a);
- the calculated *a*_M_ via eq 18c is indeed at the minimum of *N*_22_ (Figures 7b and 8b);
- *N*_22_ at Point M is between −0.78 and −0.84 for a sign of monolayer coverage (Figures 7b, 8b, and 9). The value of *N*_22_ should be quoted with STSA.

This range of *N*_22_ is the statistical thermodynamic translation of the IUPAC guideline^10^ for *C*_B_ to be between 80 and 150 for the monolayer–multilayer mechanism, which requires further work for clarification.

#### Evaluating the Underlying Assumptions via Simulation

The above procedure for surface area estimation still contains the following assumptions inherited from the BET analysis:

(a) The interface can be approximated as a planar monolayer.
(b) The standard value of the probe’s cross-sectional area is valid.

Fortunately, a key attribute of the approach here is that the above assumptions can be examined via classic molecular dynamics or Monte Carlo simulations that allow the evaluation of these key statistical thermodynamic quantities. (a) If the interface is a monolayer, the sorbate–surface correlation function has a sharp first peak and further peaks are negligibly small. For an interface that cannot be considered planar or monolayer (such as microporous materials like zeolite), reporting the Point M capacity instead of the surface area may be more realistic. (b) The effective molecular size of sorbates can be evaluated using the sorbate–sorbate distribution function. Thus, the macroscopic (the isotherm) and microscopic (i.e., molecular dynamics or Monte Carlo simulations) pictures of sorption can be linked and cross-validated.

#### Monolayer Versus Pore Filling

Extensive comparisons with simulation have revealed recently that the BET approach overestimates the surface area due to the contributions from pore filling^14^ and that distinguishing pore filling and monolayer filling is crucial for a reliable surface area determination.^15^ Addressing this difficult question requires further work. However, we would like to point out, based on our previous work on adsorption on porous materials,^21,23,24^ that *N*_22_ may still play a crucial role in connecting the gradient of an isotherm to the number of sorbates that sorb cooperatively. *N*_22_ changes with *a*_2_ (Figures 7b and 8b) and plays a central role in understanding how a macroscopic isotherm is composed of different sorption processes.

## Conclusions

Difficulties have persisted in surface area estimation using the BET analysis of an isotherm. The present paper has identified the causes of the difficulties and demonstrated how they can be overcome. Difficulties have arisen from

i trying to fit the BET model to the isotherms that break its basic assumptions; and
ii ambiguous and unclear physical meanings of the BET constant and the monolayer capacity

The introduction of the consistency criteria helped eliminate the unphysical solutions in (i) but has perpetuated (ii) by making what looks like a straightforward linear plot (the BET plot) more complicated to use. The statistical thermodynamic fluctuation theory, due to its model-free nature, has

i shown that the statistical thermodynamic isotherm, whose special and restricted case is the BET model, removes the need for force-fitting the BET model to sorption data; and
ii translated the objectives of the BET analysis into the language of the fluctuation theory.

We have removed the need for the BET model to carry out surface area estimation.

Our new, alternative approach is the generalization of the BET analysis and can be carried out without its restrictive assumptions or a force-adaptation of the BET model to reality. The key ideas are

I the excess number *N*_22_, a measure of sorbate–sorbate interaction at the interface, as central to interfacial coverage;
II Point M, at which *N*_22_ takes the minimum value, as the precise location of interfacial coverage; and
III adsorption at Point M replaces the monolayer capacity for calculating the surface area.

This new procedure for the calculation of the statistical thermodynamic surface area (STSA) can be carried out without being restricted to an isotherm model and without the consistency criteria necessitated by using a model beyond its applicability. In our statistical thermodynamic generalization of BET-based approaches, the excess number, *N*_22_, as a measure of sorbate–sorbate interaction at the interface, will play a central role

- as a replacement of the BET constant, representing the clarity of the knee and the applicability of the monolayer coverage;
- in linking the macroscopic measurement (isotherms) to microscopic (simulation) measurement, to clarify, for example, whether the interfacial filling is monolayer-like, pore-filling-like, or with a significant monolayer–multilayer overlap; and
- as a measure of sorption cooperativity, which is especially important for porous systems.

Thus, the problems of the BET analysis have been overcome by the clarity, generality, and applicability afforded by model-free statistical thermodynamics. This was brought about by a statistical thermodynamic generalization of the BET approach. However, this does not mean that our new general theory eliminates the difficulties posed by the monolayer–multilayer overlap or the ambiguity in distinguishing between monolayer coverage and pore filling. What we have achieved is to establish the general statistical thermodynamic measures for interfacial filling that do not depend on a restricted isotherm model. To address these questions, a systematic comparison with computer simulation is indispensable in conjunction with our new approach. The extension of our approach includes an examination of other approaches to surface area estimation based on different adsorption models and to clarify how the difference between the monolayer and pore-filling behaviors manifests in isotherms.

## Supporting Information Available

Negligibility of the vapor reference system; interfacial versus vapor contributions to the surface excess; vapor-interface partition function; and measuring sorption at an infinitely dilute sorbate limit (PDF)
