# Supplementary material for: Surface Area Estimation: Replacing the Brunauer–Emmett–Teller Model with the Statistical Thermodynamic Fluctuation Theory
Source: Langmuir. 2022 Jun 17;38(26):7989–8002. doi: 10.1021/acs.langmuir.2c00753 (PMC9261182; doi:10.1021/acs.langmuir.2c00753)
Supplement: Supplementary file 1 — la2c00753_si_001.pdf [file la2c00753_si_001.pdf]

Supporting information

Surface Area Estimation: Replacing the Brunauer−Emmett−Teller Model with the Statistical Thermodynamic Fluctuation Theory

Seishi Shimizu[1,*] and Nobuyuki Matubayasi[2]
[1]York Structural Biology Laboratory, Department of Chemistry, University of York, Heslington, York YO10 5DD, United Kingdom. Email: seishi.shimizu@york.ac.uk
[2]Division of Chemical Engineering, Graduate School of Engineering Science, Osaka University, Toyonaka, Osaka 560-8531, Japan

Table of Contents

Negligibility of the Vapor Reference System. p.1
Interfacial versus Vapor Contributions to the Surface Excess (Table S1). p.2
Vapor-Interface Partition Function. p.3
Measuring Sorption at Infinitely Dilute Sorbate Limit. p.4

Negligibility of the Vapor Reference System. Here we show that our statistical thermodynamic expressions of n_m and C_B (eqs 10b and 10c) can be simplified under the realistic BET parameter ranges. We will show that the contributions from the vapor reference systems are negligible, even under the dilute sorbate limit, with the common assumption that the solid part is also negligible (no absorption into the solid interior). To this end, let us introduce the vapor-interface partition coefficient at $a_2 \to 0$, as

$$K = \left(\frac{\langle n_2 \rangle}{\langle n_2^g \rangle}\right)_{a_2 \to 0} \quad \text{(S1)}$$

As will be shown in **Vapor-Interface Partition Function** section, K can be linked to the BET model parameters as

$$K - 1 = C_B \frac{\frac{n_m}{v^{(0)}}}{c_2^{\ominus}} \quad \text{(S2)}$$

where $v^{(0)}$ is the volume of the interface at $a_2 \to 0$ limit (for a simple, planar, monolayer surface, $v^{(0)}$ is simply the product of the monolayer thickness and the interfacial surface area). $\frac{n_m}{v^{(0)}}$ represents the "monolayer capacity" per interfacial volume. Note that the monolayer capacity belongs to the BET model whereas $v^{(0)}$ is a statistical thermodynamic quantity that does not involve the BET model assumptions. Now $K \gg 1$ can be shown by realizing that under a sufficiently large C_B

$$\frac{n_m}{v^{(0)}} \gg c_2^{\ominus} \quad \text{(S3)}$$

holds for common combinations of adsorbate and adsorbent, where $v^{(0)}$ is in the same order of magnitude as that of the monolayer and $c_2^{\ominus}$ is the saturated vapor concentration. Under this condition, we show that the statistical thermodynamic interpretations of C_B and n_m can be simplified. To do so, let us start from the following relationship derived in the **Vapor-Interface Partition Function** section:

$$\frac{C_B - 2}{c_2^{\ominus}} = -\frac{K^2}{K-1} G_{22}^{(0)} + \frac{G_{22}^{g}}{K-1} \quad \text{(S4)}$$

We aim to compare $\frac{C_B-2}{c_2^{\ominus}}$ and $\frac{G_{22}^{g}}{K-1}$. To do so, let us $\frac{C_B-2}{c_2^{\ominus}}$ (at $C_B = 80$) and G_{22}^{g} based on the experimental data for nitrogen at 77 K, argon at 87 K, and water at 298 K (**Table S1**). In all these cases, $\frac{C_B-2}{c_2^{\ominus}}$ is much larger in magnitude than G_{22}^{g}. Hence, under $K \gg 1$, the dominant contribution must necessarily come from the first term in eq S4, while the second term is negligible. Under this condition, eqs 9, 10b, and 10c can be simplified as

$$C_B \simeq -\left(\frac{N_{22}}{a_2}\right)_{a_2 \to 0} \quad \text{(S5)}$$

$$n_m \simeq -\left(\frac{\langle n_2 \rangle}{N_{22}}\right)_{a_2 \to 0} \quad \text{(S6)}$$

using exclusively the quantities pertaining to the interface. Note that K becomes even larger at finite a_2.

Using the sorbate-sorbate Kirkwood-Buff integral $G_{22} = N_{22}/c_2$ and $a_2 = c_2^{g}/c_2^{\ominus}$ using the concentration of saturated vapor, $c_2^{\ominus}$, eq S5 can be rewritten as

$$C_B = -K c_2^{\ominus} (G_{22})_{a_2 \to 0} \quad \text{(S7)}$$

The Kirkwood-Buff integral leads to also to the simplification of eq S6 as

$$n_I \simeq \left(\frac{v}{-G_{22}}\right)_{a_2 = a_r} \quad \text{(S8)}$$

Table S1. Interfacial versus Vapor Contributions to the Surface Excess.

	T / K	$P^{\ominus}$ / kPa	B_2 / cm^3 mol^{-1}	$c_2^{\ominus}$ / mol cm^{-3}	$(C_B - 2)/c_2^{\ominus}$ / cm^3 mol^{-1}	$G_{22}^{g} = -2B_2$ / cm^3 mol^{-1}
nitrogen	77	1.013 × 10^2	−2.78 × 10^2 [a]	1.58 × 10^{-4}	4.9 × 10^5 [c]	5.56 × 10^2
argon	87	1.013 × 10^2	−2.39 × 10^2 [b]	1.40 × 10^{-4}	5.6 × 10^5 [c]	4.78 × 10^2
water	298	3.169	−1.16 × 10^3 [c]	1.28 × 10^{-6}	6.1 × 10^7 [c]	2.33 × 10^3

[a]From Ref 1; [b]From Ref 2 at 87.2 K; [c]From Ref 3 at 300 K; [c]BET constant at $C_B = 80$. The saturation pressures were taken from Pini.[4]

Vapor-Interface Partition Function. First, we derive the $a_2 \to 0$ expression of the vapor-to-interface partition coefficient (eq S1). To do so, let us start from the $a_2 \to 0$ behavior of our statistical thermodynamic isotherm (eq 7), i.e.,

$$\langle n_2 \rangle - \langle n_2^g \rangle \simeq \frac{a_2}{A_0} \tag{S9}$$

together with the definition of the activity,

$$a_2 = \frac{\langle n_2^g \rangle / v^{(0)}}{c_2^{\ominus}} \tag{S10}$$

under ideal gas behavior. Note that the small contribution from $\langle n_2^s \rangle$ has been neglected. Therefore, from eq S10, we obtain the following limiting behavior for $\langle n_2^g \rangle$

$$\langle n_2^g \rangle = c_2^{\ominus} v^{(0)} a_2 \tag{S11}$$

From eqs S9 and S11, we obtain the following behavior at $a_2 \to 0$:

$$\langle n_2 \rangle \simeq \left(\frac{1}{A_0} + c_2^{\ominus} v^{(0)} \right) a_2 \tag{S12}$$

Therefore, from eqs S11 and S12, we obtain

$$K = \left(\frac{\langle n_2 \rangle}{\langle n_2^g \rangle} \right)_{a_2 \to 0} = 1 + \frac{1}{A_0 c_2^{\ominus} v^{(0)}} \tag{S13}$$

Using the correspondence with the BET model (eq 10b) to rewrite A_0 using the BET constants, we obtain

$$K - 1 = C_B \frac{\frac{n_m}{v^{(0)}}}{c_2^{\ominus}} \tag{S14}$$

Here, $\frac{n_m}{v^{(0)}}$ is the "monolayer capacity" per interfacial volume. The monolayer capacity belongs to the BET model whereas $v^{(0)}$ is a statistical thermodynamic quantity that does not involve the BET model.

Second, we express the BET constant C_B in terms of the vapor-to-interface partition function, K (eq S1). Equations 8, 9, and 10c, before introducing $C_B - 2 \simeq C_B$, can be rewritten in combination as

$$C_B - 2 = -\left(\frac{1}{a_2} \frac{\langle n_2 \rangle^2 G_{22} - \langle n_2^g \rangle^2 G_{22}^g}{v\left(\langle n_2 \rangle - \langle n_2^g \rangle\right)} \right)_{a_2 \to 0} \tag{S15}$$

$$= -\left(\frac{\frac{\langle n_2^g \rangle}{v}}{a_2} \right)_{a_2 \to 0} \left(\frac{K^2 G_{22} - G_{22}^g}{K - 1} \right)_{a_2 \to 0}$$

Equation S15 can be simplified using eq S10 to yield

$$C_B - 2 = -\frac{K^2 c_2^{\ominus} G_{22}^{(0)} - c_2^{\ominus} G_{22}^g}{K - 1} \tag{S16}$$

Measuring Sorption at Infinitely Dilute Sorbate Limit. Since our statistical thermodynamic theory is valid for any a_2, it can be applied to the following experimental techniques that focus on the dilute a_2 behavior. Firstly, the gas-solid virial coefficient measurements[5–7] have direct access to $G_{s2}^{(0)}$. Secondly, inverse gas chromatography at infinite dilution (IGC-ID)[8,9] also aims to probe the low activity behavior of the isotherm. Thirdly, the atomic force microscopy (AFM)[10,11] probes surface-sorbate interaction. It is important to recognize that the force in AFM is the potential of mean force, which is related to the surface-sorbate correlation function via G_{s2} in eq 8.[10,11] Therefore, the importance of the limiting $a_2 \to 0$ behavior revealed by statistical thermodynamics is common to the goals of AFM and IGC-ID.

References

(1) Schramm, B.; Gehrmann, R. Second Virial Coefficients of Nitrogen at Very Low Temperatures. *J. Chem. Soc. Faraday Trans. 1* **1979**, *75*, 479–480. https://doi.org/10.1039/F19797500479.

(2) Schramm, B.; Hebgen, U. The Second Virial Coefficient of Argon at Low Temperatures. *Chem. Phys. Lett.* **1974**, *29* (1), 137–139. https://doi.org/10.1016/0009-2614(74)80151-X.

(3) Harvey, A. H.; Lemmon, E. W. Correlation for the Second Virial Coefficient of Water. *J. Phys. Chem. Ref. Data* **2004**, *33*, 369–376. https://doi.org/10.1063/1.1587731.

(4) Pini, R. Interpretation of Net and Excess Adsorption Isotherms in Microporous Adsorbents. *Microporous Mesoporous Mater.* **2014**, *187*, 40–52. https://doi.org/10.1016/j.micromeso.2013.12.005.

(5) House, W. A.; Jaycock, M. J. The Application of the Gas–Solid Virial Expansion to Argon Absorbed on the (100) Face of Sodium Chloride. *Proc. R. Soc. London. A. Math. Phys. Sci.* **1976**, *348*, 317–337. https://doi.org/10.1098/rspa.1976.0041.

(6) Okambawa, R.; Benaddi, H.; St-Arnaud, J. M.; Bose, T. K. Gas-Solid Interaction and the Virial Description of the Adsorption of Methane on Steam-Activated Carbon. *Langmuir* **2000**, *16*, 1163–1166. https://doi.org/10.1021/la9901231.

(7) Cascarini de Torre, L. E.; Flores, E. S.; Llanos, J. L.; Bottani, E. J. Gas-Solid Potentials for N2, O2, and CO2 Adsorbed on Graphite, Amorphous Carbons, AI2O3, and TiO2. *Langmuir* **1995**, *11*, 4742–4747. https://doi.org/10.1021/la00012a027.

(8) Ho, R.; Heng, J. Y. Y. A Review of Inverse Gas Chromatography and Its Development as a Tool to Characterize Anisotropic Surface Properties of Pharmaceutical Solids. *KONA Powder Part. J.* **2012**, *30*, 164–180. https://doi.org/10.14356/kona.2013016.

(9) Jones, M. D.; Young, P.; Traini, D. The Use of Inverse Gas Chromatography for the Study of Lactose and Pharmaceutical Materials Used in Dry Powder Inhalers. *Adv. Drug Deliv. Rev.* **2012**, *64*, 285–293. https://doi.org/10.1016/j.addr.2011.12.015.

(10) Gullingsrud, J. R.; Braun, R.; Schulten, K. Reconstructing Potentials of Mean Force through Time Series Analysis of Steered Molecular Dynamics Simulations. *J. Comput. Phys.* **1999**, *151*, 190–211. https://doi.org/10.1006/jcph.1999.6218.

(11) De Pablo, P. J. Introduction to Atomic Force Microscopy. *Methods Mol. Biol.* **2011**, *783*, 197–212. https://doi.org/10.1007/978-1-61779-282-3_11.
